# Exploratory Qualitative Study of Fire Preparedness Among High-rise Building Residents

**DOI:** 10.1371/currents.dis.aa27444baa486dc3d5b3fa7c28009b22

**Published:** 2018-08-31

**Authors:** Gary Glauberman, Kristine Qureshi

**Affiliations:** University of Hawaii at Manoa School of Nursing and Dental Hygiene, Honolulu, Hawaii, USA; Global Health Nursing, School of Nursing and Dental Hygiene, University of Hawaii at Manoa, Honolulu, Hawaii, USA

## Abstract

Introduction: Fire hazards are an extreme risk to occupants of high-rise buildings. Little attention has been paid to emergency and evacuation preparedness among people living in high-rise buildings. This paper reports on emergency fire preparedness among residents of a high-rise building that has experienced multiple fires in the past.

Methods: An exploratory qualitative pilot study was conducted using key informant interviews. Six residents participated. Themes on preparedness for fires and emergency evacuation were extracted.

Results: Findings indicated varying levels of preparedness for fires and emergency evacuation among residents. Factors influencing residents’ emergency preparedness included fire risk perception, owner or renter status, and building-level emergency preparedness. Fire alarms were considered to be an ineffective evacuation cue. Severe cues such as seeing fire or smoke were more likely to prompt evacuation. Participants provided a series of suggestions to keep high-rise residents safe during fire emergencies.

Discussion: The study revealed fire preparedness knowledge, decision-making processes, and actual behaviors of residential high-rise occupants who experienced a fire emergency in their building. Main findings of the study are discussed in two themes: influences on fire emergency and evacuation preparedness, and evacuation decision-making and response to fire. Results from this pilot study will be used as the basis for a follow up study involving residents from multiple high-rise buildings.

Keywords: disaster, emergency preparedness, evacuation, fire, hazard, high-rise building, pilot study, qualitative research

## Exploratory qualitative study of fire preparedness among high-rise building residents

Currently, over half of the world’s population lives in urban areas, a proportion expected to rise to two-thirds by 2050.^1^ This global trend portends an increasing number of urban disasters.^2^ Sustained growth in urban centers is leading to higher concentrations of high-rise buildings, and an amplified risk to people living and working in these structures in the event of an emergency.

Fire hazards are an extreme risk to occupants of high-rise buildings. Between 2009 and 2013, U.S. fire departments responded to an estimated 14,500 structure fires in high-rise buildings per year. The majority of these incidences (62%) occurred in apartments and other multi-family housing. During this period, there was an annual average of 40 civilian deaths, 520 injuries, and $154 million in direct fire damage.^3^ Specific populations are much more vulnerable to such emergencies, namely the elderly, children, and people with disabilities or other health conditions that impair mobility. Deaths among older adults (> 65 years old) and very young children (< 5 years old) in residential fires are disproportionately higher to their proportion of the U.S. population.^4^ Persons with mobility impairments are also at a higher risk for injury and death during residential fires.^5^

Engaging in emergency preparedness is an effective way to reduce the risk of death or injury during crises.^6^ Factors influencing household and community emergency preparedness have been the focus of many research studies.^7^^, ^8 Little attention has been paid to specific factors influencing emergency preparedness for fires in high-rise buildings. Most of what is known on this topic has arisen from research involving office or commercial buildings. Less is known about fire preparedness among people living in residential high-rises. An exploratory qualitative pilot study was conducted to examine fire and evacuation preparedness among residents of a high-rise building that has experienced multiple fire emergencies in the past. Findings from this study are shared in this article, including specific factors influencing high-rise building occupant fire and evacuation preparedness, cues for evacuation, and suggestions to keep residents safe during actual fire emergencies.

**All-hazards preparedness in high-rise buildings**

High-rise buildings are defined by the National Fire Protection Association^3^ as buildings greater than 23 m (75 feet) in height from the ground level to the highest floor. The main uses for this type of building are residential (apartments, dormitories or other multi-family housing), office buildings, hotels, and facilities that care for the sick.^3^ Practicing all-hazards emergency preparedness can improve the likelihood that building occupants remain safe during disasters such as fires and other crises. An all-hazards approach to emergency preparedness “addresses a full range of threats and hazards, including natural, human-caused, and technology-caused.”^9^ According to the Federal Emergency Management Agency (FEMA)^10^, basic preparedness strategies common for all types of disasters include getting information about hazards and emergencies affecting families in a given location, developing an emergency plan, assembling a disaster supply kit, learning where to seek shelter from all types of disaster, identifying warning systems and evacuation routes, and practicing and maintaining an emergency plan.

High-rise building residents encounter environmental elements that make planning for all-hazards emergency preparedness unique. Services that people in high-rise buildings depend upon, such as water, electrical, transportation and communication systems, can be disrupted if a disaster damages or destroys critical infrastructure. High-rise building residents must also be able to evacuate quickly in emergency situations. Residents may have to travel long distances to get from the area of residence to safety, especially if they reside at higher elevations. The route to safety may be crowded with others trying to evacuate at the same time. Evacuation routes may be more challenging to navigate for persons with disabilities or other health conditions that impair mobility. During emergencies, evacuation routes may become too dangerous, so residents must also know how to safely shelter in place until first responders arrive.

**Commercial high-rise building emergency preparedness**

Studies on the evacuation of the World Trade Center (WTC) buildings following the September 11, 2001 terrorist attacks have provided significant understanding of factors influencing preparedness for emergency and evacuation of commercial high-rise buildings under extreme conditions.^11^^, ^12 13^, ^14 Research on incidences of high-rise building fires and terrorist attacks has also been informative in describing building occupants’ preparedness for emergencies and evacuation behavior.^15^^, ^16^, ^17 One study developed the Emergency Preparedness Knowledge and Experience Scale, to measure building familiarity and knowledge among WTC evacuees.^18^ Evacuees scored low on the scale, signifying low levels of knowledge regarding the location of building fire stairs, how to enter the stairwell, or where the stairs lead. Occupants were also unfamiliar with building evacuation plans, were not confident evacuating the building without instruction, or lacked knowledge regarding evacuation routes. While most had experience participating in emergency drills, participants reported not having experience actually entering stairwells or exiting the building during evacuation drills, or were not aware that stairwell doors were locked from the inside, preventing reentry from the stairwells on certain floors. Proulx and Reid^17^ found similarly low levels of knowledge regarding emergency procedures among evacuees of a high-rise office building fire. Other studies have shown higher levels of emergency preparedness among commercial high-rise building occupants.^16^^, ^17^, ^19 These studies reported that most participants knew the location of exit doors and/or had knowledge of building emergency evacuation plans. Participants also reported high levels of participation in some form of emergency preparedness training, such as receiving information on emergency procedures and participating in evacuation drills.

**Residential high-rise building emergency preparedness**

In a study comparing occupants of residential and commercial high-rise buildings, Zmud^19^ found that residential high-rise occupants had a lower degree of emergency preparedness knowledge than people in commercial high-rise buildings. In comparison to commercial high-rise building occupants, residential building occupants were less aware of building evacuation procedures and less likely to participate in fire drills. They also lacked awareness of occupant emergency training, or building fire safety features, such as fire pull stations, fire exits, or the building public address (PA) system. In another study that surveyed residential high-rise dwellers in Egypt, participants reported high levels of knowledge regarding their buildings’ emergency plans and locations of emergency exits. However, most participants in the study hadn’t received fire safety training and were not sure what to do in fire situations.^20^

**Methods**

**Study design and sample**

An exploratory qualitative pilot study was conducted to improve understanding of the knowledge and attitudes regarding fire and evacuation preparedness among people living in residential high-rise buildings. The study was approved by the University of Hawaii Human Studies Program as exempt from federal regulation pertaining to the protection of human research participants.

All study participants were residents of a high-rise building that had experienced multiple fires in the past. Three fires had occurred in the building within a span of seven years. Two of the building’s previous fires, one on the 8th floor and another on the 23rd floor, had been confined to the rooms of origin. The most recent fire in the building had occurred seven weeks prior to the start of the study. This fire originated on the 26th floor, and spread to multiple units on three floors of the building.

Six participants were included in this study. Study participants were identified using a snowball sampling technique, and invited to participate in-person, or were contacted by phone or email. Inclusion criteria included English-speaking adults age 18 years or older who were able to provide informed consent and had experience living in a residential high-rise building. Each person was provided information about the study’s purpose, methods, human subjects protections in place, and eligibility criteria prior to participation. Oral consent to join the study was obtained from each participant.

**Data collection and analysis**

A semi-structured interview guide was developed using information on emergency and evacuation preparedness in high-rise buildings. Interviews lasted an average of 60 minutes. All of the interviews were conducted in-person from September to October 2017. During each session, one researcher would ask questions, another would take handwritten notes. Handwritten notes were summarized and read back to participants intermittently to check for accuracy. Each session opened with an uninterrupted narrative where participants shared their experiences during the building’s most recent fire. The semi-structured interview guide was used to hone discussion on specific aspects of interest that had not been previously mentioned.

A short demographic information sheet was used to collect information about each participant’s age, gender, number of years living in high-rise buildings, floor of residency during the most recent fire, and owner/renter status. Unique code numbers were assigned to each participant. No personal identifiers of any kind were noted. All study materials were stored in password-protected files on an encrypted computer only available to the research team.

Qualitative thematic analysis^21^ was used to identify themes that emerged from the data related to participant preparedness for, and actual experience during building emergencies. Handwritten notes were reread several times and initially coded. Codes were sorted and combined to generate overarching themes and subthemes. Themes were reviewed and refined to assure they accurately reflected the data set as a whole. Strategies for establishing the trustworthiness of the study were used to maintain methodological rigor.^22^ Handwritten notes were read back to participants as a form of member checking. Two researchers reviewed and coded the handwritten notes independently at first, then compared codes with each other. Differences in codes were discussed and mutually resolved. Researchers kept the raw data and analysis to ensure an audit trail.

**Results**

**Participant demographic information**

Demographic data collected from participants of the study are summarized in Table 1. Of the six participants, two were male and four were female. The age of participants ranged from 24 to 77 years old. Three participants were owners and three were renters. Three participants lived between the 8th and 19th floors; the other three lived between the 20th and 35th floors. The number of years that participants lived in their current building ranged from 1 to 35 years. The total number of years that participants lived in any high-rise building, including their current building, ranged from 4 to 49 years.

**Table 1:** Characteristics of study participants (N=6)

Characteristic	n (%)
Gender	
Male	2 (33)
Female	4 (67)
Age	
20-39	1 (17)
40-59	2 (33)
60-80	3 (50)
Tenure	
Owner	3 (50)
Renter	3 (50)
Floor	
0-7	0 (0)
8-19	3 (50)
20-35	3 (50)
Years lived in current high-rise building	
0-5	2 (33)
6-10	2 (33)
11-15	1 (17)
>15	1 (17)
Total years lived in high-rise buildings	
0-5	1 (17)
6-10	1 (17)
11-20	2 (33)
>20	2 (33)

**Qualitative results**

Qualitative findings have been organized into four sections: 1) pre-incident, 2) peri-incident, 3) post-incident phases of the fire, and 4) a summary of participants’ suggestions for improving residential high-rise building preparedness for fire emergencies.

**Pre-incident**

**Preparedness for fire emergencies**. Participants described varying levels of household fire preparedness. Some participants had multiple fire extinguishers and smoke alarms in their apartment unit. Others had smoke alarms but no fire extinguisher, or neither of these items. All participants knew the location of the emergency exit staircases and had experience entering the stairwells in the past. Some also had actual experience evacuating the building during two fire emergencies in the past. One participant who had experienced three fires in the building maintained a high level of evacuation preparedness, stating “*I keep my purse in the foyer, always by the door*… ” One participant believed a generational difference in “fire literacy” exists, with the current generation lacking a basic understanding of fire safety, stating, “*Earlier generations knew how to manage fire…everyone had a fire plan*.”

**Fire risk perception.** Risk perception of fire among participants was generally low despite most having previous experience with fire in the building. One participant, who had experienced one fire in the building, described never having thought about fire hazards, stating, “*Before the* [most recent] *fire, we didn't have a plan for fire… never considered fire in the building, sprinklers, or proximity to the stairs.*” Among participants who were worried about fire, the risk of fire did not outweigh the benefit of building amenities, as one participant stated, “*When we bought the unit, I wanted fire sprinklers, but* [my spouse] *wanted guest parking*.” There was also a perception that fires are inevitable, as one person stated, “*One fire every 10 years is expected in a community of this* [building’s] *size*.”

**Owner/renter perspectives on fire safety responsibility.** A difference between owners and renters’ perception of responsibility for fire safety emerged from the data. Owners felt that renters expected apartment unit owners to be responsible for fire safety and equipment. An owner stated, “*Owners put* [fire extinguishers] *in units they own. Renters abdicate* [responsibility] *to owners.*” Conversely, renters described feeling responsible for the residence but felt excluded from building decisions regarding fire safety and preparedness. One renter stated, “*Renters have no voice*.” One participant didn't see any difference between renters and owners, stating, “*All are equally fire illiterate*.”

**Ineffectiveness of fire alarms as an evacuation cue.** Participants provided multiple reasons why they believed the building’s fire alarm was not an effective mechanism for initiating evacuation. Past experiences with false alarms resulted in many residents not initiating evacuation immediately after hearing the fire alarm. One participant said, “*I heard the alarm before, but did not react…it would go on for a long time*.” Another said, “*At times of false alarms, security would announce it was a false alarm with a bullhorn*.” Participants also described the difficulty in hearing alarms, or not being able to distinguish the alarm from other sounds. One person said, “*I called security to check… usually, the building alarm was faint… it was like, ‘Oh, is that the fire alarm?*’” Another person said, “*I wouldn’t hear the alarm because of multiple* [emergency vehicle] *sirens from the street*."

**Building-level fire preparedness.** Participants described an overall low level of building fire safety culture. One participant stated, “*Never had a drill… no fire safety pamphlet*.” Participants were all aware that the building did not have fire sprinklers. There was uncertainty regarding whether or not the building had a fire safety plan for persons with disabilities, or whether the building possessed a list of people who would need assistance evacuating. One participant described observing confusion about how to evacuate residents with mobility impairments during each fire that had occurred in the building. The participant described seeing people with assistive devices (wheelchairs/walkers) moving towards the stairwells but not knowing what to do when they got there, remarking, “*All* [fires] *were the same… people trying to go down the stairs with walkers… a family with a female in a wheelchair, blocking the stairwell exit… People do not know how to exit with a wheelchair*.”

**Preparedness for other types of disasters.** Varying levels of household preparedness for other types of disasters were also noted. One participant stated, “*We’re better with hurricane preparedness*.” Emergency stockpiles of supplies varied, from having a full week of food/water and supplies (flashlight, batteries), to having only limited food and water, to having no emergency supplies at all. The occupation of one participant’s spouse was very influential to family emergency preparedness. This individual’s household was well-stocked with emergency supplies and had a well-developed family reunification plan that included more than one meeting place. Another individual described being prepared for riot and civil insurrection and had listed weapons and a military-grade gas mask among the items included in an emergency supply kit.

Reasons cited for low levels of household preparedness included a lack of storage space in the apartment unit and plans for sheltering at a relative’s home (rather than in their own apartment) in the event of a natural disaster. In describing personal preparedness for disaster, one participant stated, “*Not well. If anything* [we] *keep cases of water in the closet for hurricanes, random canned goods, none in storage*… [It’s] *hard in an apartment building, there’s limited storage*.” Another participant described similar preparedness for disasters, stating, “*Nothing… Plan to go to mom’s* [house] *during hurricanes*.”

**Peri-incident**

**Evacuation cues.** Two types of cues for evacuation emerged from the data: speculative cues and severe cues. Speculative cues were those that prompted residents to seek more information but did not immediately prompt evacuation. Such cues included hearing the building fire alarm, emergency vehicle sirens, and seeing emergency vehicles (fire engines/helicopters) and equipment. One person who had experienced three fires in the building said, “*I saw a helicopter… thought it was unusual.*” Severe cues included seeing or smelling smoke, seeing fire/flames, hearing people screaming, or hearing commands to evacuate. One person said, “*I heard the alarm go off… Looked out the window and could see fire, heard screaming… We all started heading towards the door*.” Another person remarked, “*I heard the alarm, waited three minutes, opened the door and smelled smoke… My neighbor was running, knocking on doors. Lights were out in the hallway past the elevator.*”

**Evacuation process.** Initiation of evacuation was delayed by the gathering of supplies prior to moving toward the stairs. One person said, “*I went into ‘auto-mode,’ gathered computers, purse… went to the stairs.*” Movement down the stairwells was described as very orderly. During the evacuation process, people provided assistance to others who needed help down the stairs, such as older residents or persons with disabilities. One person said, “*I saw an elderly female in a bathrobe, frenzied, dazed… went into ‘take-care mode’ to care for her… helped her contact her husband*.” Another person said, “*Whenever I have to down the stairs* [during a fire], *I go slower… help others*.”

**Reliability of fire warning systems.** Despite the building having a smartphone app capable of sending push-notifications to residents, no notifications were received during the fire incident. Participants described waiting for messages on their phone about what was happening, though none came. One person said, “*I did not receive notification… called* [spouse], *he didn't get a notification on the app*…”

**Post-incident**

**Danger, stress, and uncertainty.** Participants described strong feelings of danger, stress, and uncertainty during the immediate period following evacuation. One person said, “*While on the ground, things were falling off the building… glass, debris, sides of the building*.” Another person said, “*People stood in the grassy area looking at the burning building. Not sure… was this safe? Smoke, soot, fire, glass, metal… Was there asbestos in the smoke? Was lead being melted in burning paint?*” One person noted residents seemed to be in shock, remarking, “*Many people were in a ‘zombie state.’*” During the event, participants felt stress being separated from their family members. One person said, “*I went into ‘stress/panic mode.’ I called friends… got daughter reunited with the family, then felt better.*”

Feelings of stress and uncertainty continued during the weeks and months following the fire, driven by concerns about the costs of the damage, conflicting messages regarding hazard mitigation, and worry about health and the safety of their environment. Participants described fear of exposure to hazardous materials and byproducts of the fire, such as asbestos, mold and lead paint. One participant shared, “*We were not sure the unit was safe… worried about asbestos coming from vent… we decided to cover the vents in the apartment.*” One participant stated, “*I feel like I’m experiencing PTSD*.” The participant suspected a young child in the family might also be similarly affected, remarking, “*My son said, ‘I don’t want you to die in a fire.’*” Another participant whose apartment was not damaged in the most recent fire described his feelings as, “*Survivor guilt…why was I spared?*”

**Recovery activities.** Immediately following the event, people took action toward their own recovery. Pictures of damaged property were taken for insurance purposes and then discarded. One person said, “*We were not sure what to keep/discard. We took video of water damage. Saw black soot on shower bathroom walls. Not sure to keep or wash down*.” Participants also shared their children’s reactions to the incident. One participant said, “*After, in a hotel my son said, ‘I feel safe now.*’” Another participant realized her young child needed to continue playing despite disaster recovery activities, remarking, “*I put blue tape *[on the carpet]* to tell my son don’t come inside* [damaged areas]. *I saw my son playing with a small item as a toy*.”

**Social support.** Residents, family members, and community organizations provided support to people affected by the fire. Friends and family provided displaced residents with temporary housing. Community agencies provided residents with shelter and supplies. One person said, “*The next morning* [after the fire], *food, donations, clothes, supplies were donated… it was uplifting.*” Survivors of the event were giving and receiving support from each other. One person said, “*Those in need were helping others. The building, as a community, all came together*.”

**Impact on future fire and emergency preparedness.** Impact on preparedness for emergencies among participants was mixed. Most stated that they had made no changes to their personal/family fire or emergency preparedness since the fire. One person remarked, “*After the fire, I made a list… but it’s still just chaos.*” Some participants described intentions to increase their family’s emergency preparedness supply stockpile, updating their family emergency plans for future disasters, or seeking out training on fire safety. One participant said, “[Before the fire], *we had one week worth of water and food supply… Will increase to 14 days.* [My spouse is] *now talking about where to meet when tsunami hit* [sic].”

**Suggestions to prepare high-rise residents for fire emergencies**

Participants provided suggestions regarding how to better prepare residents of high-rise buildings for fire emergencies. Suggestions were organized into three levels: individual/family, building management/condo association, and systems (community/public policy) levels.

**Individual/family level. **Participants’ suggestions for individuals/families pertained to evacuation, basic fire safety, and health impact of hazardous material exposure (Table 2).

**Table 2: Individual/ family level suggestions for residential high-rise fire preparedness:** 

Category	Suggestions
Know how to evacuate	• Develop household evacuation plan. Include the route to safety/what items to bring • Place copy of the plan on the entryway door • Close widows prior to evacuating • Knock on neighbors’ doors while evacuating • Consider using hanging ladders to evacuate lower floor balconies
Practice basic fire safety	• Install/maintain apartment smoke alarms • Practice proper fire extinguisher use • Unplug appliances when not in use • Close windows/doors when leaving apartment
Know health impact of fire events	• Learn signs/symptoms of exposure to hazardous materials

***Know how to evacuate.*** Residents should have a plan to evacuate the building that all members of a household are aware of. The plan should be written and placed on the back of the door. The plan should include what route to take to safety, and what items to bring when evacuating, such as a flashlight, water, phone and charger, and any important medications. Prior to evacuating, residents should quickly close their windows to protect their unit from smoke or water damage. While evacuating, people should knock on their neighbors’ doors to warn them to get out. Residents of lower floors should consider using hanging ladders to evacuate from their balconies.

***Practice basic fire safety.*** All occupants should have smoke alarms and at least one fire extinguisher in their residence. They should replace smoke alarm batteries annually. Proper use of the fire extinguisher should be practiced. Appliances should be unplugged when not in use. Windows and doors should be kept closed when leaving the apartment.

***Know the impact of the event on health.*** People should be aware of and be able to recognize adverse health effects resulting from exposure to hazardous materials in a building affected by a fire. These include exposure to smoke, mold, asbestos, and other hazardous byproducts of a fire.

**Building management/condo association level. **Suggestions for the building include improving building fire safety systems, emergency communications, fire drills and training, emergency planning, and social support systems (Table 3).

**Table 3: Building management/condo association level suggestions for residential high-rise fire preparedness:** 

Category	Suggestions
Enhance building fire safety systems	• Place fire extinguishers in every kitchen • Install fire sprinklers, automatically-closing doors, illuminate pathways to exits, and audible fire alarms • Install pumps to fill standpipes prior to the fire department arriving
Robust emergency communication system	• Develop an emergency communications plan that designates how warning messages are sent, and frequency/timing of messages • Messages should provide clear instructions on what to do/whether to evacuate • Establish multiple channels for communicating emergencies • Ensure communication system redundancy
Building drills/training	• Hold annual fire drills • Provide voluntary fire safety training to residents • Schedule fire alarm testing so that residents can check whether alarms are audible • Develop building self-sufficiency so that residents can extinguish fires prior to the fire department arriving
Develop plans to keep all residents safe	• Distribute pamphlets describing building fire safety and evacuation procedures • Designate fire captains on each floor who know which residents require evacuation assistance • Mark doors to signify residents needing evacuation assistance • Allow renter representation on the building’s governing board • Establish emergency vehicle access for all sides of the building without immediate roadway access
Establish a building social support network	• Hold social events to foster a sense of community within the building • Develop a method for following up with residents after an emergency

***Improve building fire safety systems.*** Participants recommended enhancing building fire safety by installing fire sprinklers, doors that close automatically in the event of a fire, and an emergency lighting system to illuminate pathways to the exits. Fire alarms should be audible and should automatically alert building management/security personnel. Fire extinguishers should be placed in the kitchen of every apartment in the building. The building should also consider installing pumps to fill standpipes prior to the arrival of the fire department.

***Robust emergency communication system.*** Multiple methods for communicating emergencies to residents should be in place. Suggestions included installation of a PA system, a reliable mobile alert system, and a TV broadcast warning system. Warning systems should alert residents of emergency situations, and instruct them whether evacuation is necessary. The building should develop an emergency communications plan that designates which building personnel is responsible for sending out warning messages, how messages can be sent, what information the messages should contain, and the frequency and timing of such messaging. The plan should include redundancies in case portions of the building (or management staff) are not accessible or available.

***Building drills and training.*** Fire drills should be held once or twice a year. Building management should partner with the fire department to provide residents with voluntary training on proper use of fire safety equipment. Residents should develop self-sufficiency for fighting fires prior to the arrival of firefighters. Fire alarm testing should be scheduled so that residents can check if alarms are audible from all rooms of the apartment.

***Develop plans to keep all residents safe.*** A pamphlet describing fire safety and evacuation procedures should be made available to residents. Special considerations should be arranged for persons with disabilities such as designating a fire captain on each floor who knows which residents require assistance evacuating. Doors could also be marked to signify the residence of someone who needs assistance. Renters should have representation on the building’s governing board to provide diverse input. Emergency vehicle access should be established for all sides of the building, including sides of the building that do not have immediate roadway access.

***Establish building social support network.*** Building management should organize social events to foster a sense of community within the building in periods prior to and following an emergency. A method should be in place for following up with persons suffering from post-traumatic stress disorder (PTSD). Networks for post-disaster support should also be established to provide affected families with resources such as temporary housing, psychological counseling, and interpretation services.

**System (community/public policy) level. **Wider changes to policy and education regarding fire safety were also suggested (Table 4).

**Table 4: System (community/public policy) level suggestions for residential high-rise fire preparedness:** 

Category	Suggestions
Policy suggestions	• Mandate annual fire drills • Conduct regular fire safety inspections • Enforce fire safety rules
Education	• Improve community fire safety education • Provide school children with fire safety education, including how to use fire extinguishers.
Social support network	• Develop community networks to support families post-disaster

Laws should be created to mandate fire drills at least twice a year in residential high-rise buildings. Fire safety inspections should be conducted regularly, and safety rules should be strongly enforced. Finally, more should be done to better educate the population about basic fire safety. Specifically, elementary school children should receive fire safety education and be taught how to properly use fire extinguishers.

**Discussion**

Findings from this exploratory pilot study offer valuable insight into emergency fire and evacuation preparedness among residents of a high-rise building that experienced multiple fires. The main findings of the study are discussed in two themes: influences on fire and evacuation preparedness, and evacuation decision-making and response to fire.

**Influences on fire and evacuation preparedness**

Risk awareness, critical awareness of disasters, and hazard anxiety are required to motivate preparedness for disasters.^6^ Overall, all participants had some knowledge about emergency preparedness, and half had actually taken some action to prepare for disasters. Some participants described being more prepared for hurricanes than a building fire. Participants seemed aware of the risk for fire in the building, yet there still existed a low level of anxiety regarding fire hazards among some participants. Not all participants were aware that the building lacked a fire sprinkler system prior to the most recent fire. The building was built before fire sprinkler systems were mandated by local building codes. Further inquiry into beliefs about emergency fire preparedness and hazard risk among high-rise building residents is recommended to better understand these findings.

All participants described limited support from the building management or owners’ association for communal fire preparedness efforts. Participants of this study, some who had lived in the building for many years, were not aware of any fire drills, or other types of fire preparedness activities ever having been conducted in their building. This lack of emphasis on fire preparedness at the building level may have influenced household fire preparedness among study participants. This is analogous to findings from studies done in commercial high-rise buildings. For example, Gershon et al.^18^ found that WTC evacuees who reported lower organizational support for emergency preparedness and response capabilities on their floors were less knowledgeable about building fire safety features and evacuation procedures, and took longer to evacuate the building during the September 11, 2001, WTC terrorist attacks. The influence of residential building association emergency preparedness safety culture on household preparedness is not well documented, and further research on this topic is recommended.

Differences in fire preparedness among renters and owners were noted in terms of perceived responsibility for preparedness and actual fire preparedness behaviors. Previous studies have described multiple factors affecting renters’ and owners’ preparedness for natural and technological disasters, including access to resources, exposure to hazard information, differences in motivation, and perceived responsibility for preparedness.^23^ ^24^ Findings from this study suggest similar factors may play a role in fire preparedness in residential high-rise buildings. The three participants who were owners tended to be more prepared than the renters for fires and other disasters. One participant who was a renter stated that the inability to participate in condominium board meetings precluded this individual’s ability to provide input into building safety matters. This resulted in a feeling of exclusion regarding important building fire safety decisions. Previous studies have reported that persons who believed that they had less choice in preparedness matters were less likely to prepare for natural disasters.^24^ Findings from this study suggest that similar factors may also influence personal fire preparedness of high-rise building renters.

**Evacuation decision-making and response to fire**

Previous studies on commercial high-rise building evacuation have described factors influencing evacuation decision-making among occupants. These studies have shown that environmental cues, if perceived as indicating the existence of a threat, can interrupt an individual’s normal activities, and influence protective action. Individuals will either seek additional information, engage in actions to protect people or property, perform actions to reduce psychological stresses, or resume normal activities. In order for an individual to perceive the existence of a threat, the individual must first receive cue(s), pay attention to the cue(s), and comprehend the cue(s).^25^^, ^26

In this study, participants described their inability to hear the fire alarm clearly or discern it from other noises. Furthermore, participants had experienced multiple false alarms in the past. The sounding of the fire alarm was not perceived as a cue that a true fire threat existed. Rather, upon hearing the fire alarm, participants sought more information (listening or waiting for more cues) or did nothing (continuing what they were doing). This behavior is a common reaction to building fires. Building occupants often ignore fire alarms because they fail to hear the signal, fail to recognize the signal as a fire alarm, or have lost confidence in the system due to nuisance or false alarms.^27^

Upon seeking and receiving more extreme cues, such as seeing smoke or flames, or hearing screaming, participants were more likely to perceive the presence of a real fire threat and began to take protective action. Participants engaged in a variety of activities to protect people or property, such as gathering possessions, assembling family members and neighbors, and moving towards the stairs. These actions are consistent with what is known regarding human behavior during fires. Occupants are more likely to interpret a situation as a fire when they are presented with a higher number of cues, a consistent set of cues, and unambiguous cues.^28^

Fire drills and fire safety education can help occupants better judge a fire situation, understand the importance of quick evacuation, and become more familiarized with exit routes.^29^ Findings from this study indicate a need (and desire) among high-rise residents for fire safety education and training. Participants in this study described a period of 3 or more minutes of seeking further cues after hearing the fire alarm sound. Such delays in initiating evacuation increase the risk of injury or death in fire situations. Providing training and education on fire and evacuation preparedness should improve high-rise residents’ ability to quickly recognize emergency fire situations, decrease the amount of time it takes to initiate and complete evacuation, and potentially save lives.

**Limitations and conclusion**

This study involved a small number of participants from one residential high-rise building. Transferability of the findings to other residential high-rise buildings may be limited. Furthermore, findings may not pertain to emergency preparedness for other types of high-rise buildings, such as commercial office buildings or hotels. Additionally, one member of the research team was a resident of the building from which participants were recruited. Some participants knew the researcher prior to the study, which may have introduced some bias into the study’s findings. Conversely, as a result of knowing the researcher, participants may have been more open in sharing their experiences. Despite these limitations, this study contributes new knowledge on the subject of fire and evacuation preparedness among people living in high-rise buildings and highlights multiple areas for further research. Findings from this study will be used as the basis for a future study involving more participants from multiple residential high-rise buildings. It is hoped that further study on this topic will contribute to policies and programs that improve residential high-rise building fire safety.

## Data Availability

Data available upon request to the University of Hawaii Human Studies Program.

Contact information: University of Hawaii Human Studies Program, 1960 East-West Road, Biomedical Sciences Building B104, Honolulu, HI 96822 ( email: uhirb@hawaii.edu, phone: +1(808) 956-5007 )

Publicly posting the data might reveal the identity or location of participants. This is because the transcripts of the qualitative key informant interview data collected during interviews contain multiple specific references to the building name/location, names of surrounding community features (parks, schools) and even the floors of the building that participants live(d) on in the building. Therefore, it is not appropriate to publicly post qualitative key informant interview transcript data. Request for this data can be done through a request from the University of Hawaii Human Studies Program.

## Competing Interests

The authors have declared that no competing interests exist.

## Corresponding Author

The corresponding author is GARY GLAUBERMAN (glauberm@hawaii.edu)

## References

[1] United Nations Department of Economic and Social Affairs. (2014). World urbanization prospects: The 2014 revision, highlights. Retrieved from https://esa.un.org/unpd/wup/publications/files/wup2014-highlights.pdf

[2] United Nations Human Settlements Program. (2007). Enhancing urban safety and security: Global report on human settlements 2007. Retrieved from http://www.citiesalliance.org/sites/citiesalliance.org/files/UNH-EnhancingUrbanSafety.pdf

[3] National Fire Protection Association. (2016). High-rise building fires. Retrieved from http://www.nfpa.org/~/media/files/news-and-research/fire-statistics/occupancies/oshighrise.pdf

[4] Gilbert, S. W., & Butry, D. T. (2017). Identifying vulnerable populations to death and injuries from residential fires. Injury prevention, 423-443 10.1136/injuryprev-2017-042343 28774896

[5] Rooney, C., & White, G. W. (2007). Consumer Perspective. Journal of Disability Policy Studies, 17(4), 206-215.

[6] Paton, D. (2003). Disaster preparedness: A social-cognitive perspective. Disaster Prevention and Management: An International Journal, 12(3), 210-216.

[7] Paton, D., Bajek, R., Okada, N., & McIvor, D. (2010). Predicting community earthquake preparedness: A cross-cultural comparison of Japan and New Zealand. Natural Hazards, 54(3), 765-781.

[8] Thomas, T. N., Leander-Griffith, M., Harp, V., & Cioffi, J. P. (2015). Influences of preparedness knowledge and beliefs on household disaster preparedness. MMWR: Morbidity and Mortality Weekly Report, 64(35), 965-971. 10.15585/mmwr.mm6435a2 26356729

[9] National Fire Protection Association. (2013). NFPA 1600: Standard on disaster/emergency management and business continuity programs. Quincy, MA.

[10] Federal Emergency Management Agency. (2004). Are you ready? An in-depth guide to citizen preparedness. Retrieved from https://www.fema.gov/pdf/areyouready/areyouready_full.pdf

[11] Gershon, R., Qureshi, K., Rubin, M., & Raveis, V. (2007). Factors associated with high-rise evacuation: Qualitative results from the World Trade Center Evacuation. Prehospital & Disaster Medicine, 22(3), 165-173. 10.1017/s1049023x0000460x 17894208

[12] Gershon, R., Rubin, M., Qureshi, K., Canton, A., & Matzner, F. (2008). Participatory action research methodology in disaster research: Results from the World Trade Center evacuation study. Disaster Med Public Health Prep, 2(3), 142-149. 10.1097/DMP.0b013e318184b48f 18845929

[13] Kuligowski, E., & Mileti, D. (2009). Modeling pre-evacuation delay by occupants in World Trade Center Towers 1 and 2 on September 11, 2001. Fire Safety Journal, 44(4), 487-496.

[14] Shields, T. J., Boyce, K. E., & McConnell, N. (2009). The behaviour and evacuation experiences of WTC 9/11 evacuees with self-designated mobility impairments. Fire Safety Journal, 44(6), 881-893.

[15] Glenshaw, M. T., Vernick, J. S., Li, G., Sorock, G. S., Brown, S., & Mallonee, S. (2007). Preventing fatalities in building bombings: What can we learn from the Oklahoma City bombing? Disaster Med Public Health Prep, 1(1), 27-31; discussion 31-23. 10.1097/DMP.0b013e3180640cd7 18388599

[16] Kuligowski, E., & Hoskins, B. (2010). Occupant behavior in a high-rise office building fire – NIST technical note 1664. Gaithersburg, MD: National Institute of Standards and Technology.

[17] Proulx, G., & Reid, I. (2006). Occupant behavior and evacuation during the Chicago Cook County Administration Building Fire. Journal of Fire Protection Engineering, 16(4), 283-309.

[18] Gershon, R., Magda, L., Riley, H., & Sherman, M. (2012). The World Trade Center evacuation study: Factors associated with initiation and length of time for evacuation. Fire and Materials, 36(5-6), 481-500.

[19] Zmud, M. (2008). Public perceptions of high-rise building emergency evacuation preparedness. Fire Technol, 44(4), 329-336.

[20] Gerges, M., Mayouf, M., Rumley, P., & Moore, D. (2017). Human behaviour under fire situations in high-rise residential building. International Journal of Building Pathology and Adaptation, 35(1), 90-106.

[21] Braun, V., & Clarke, V. (2006). Using thematic analysis in psychology. Qualitative research in psychology, 3(2), 77-101.

[22] Lincoln, Y. S., & Guba, E. G. (1985). Naturalistic inquiry (Vol. 75). Beverly Hills, CA: Sage Publications.

[23] Burby, R. J., Steinberg, L. J., & Basolo, V. (2003). The tenure trap: The vulnerability of renters to joint natural and technological disasters. Urban Affairs Review, 39(1), 32-58.

[24] Mulilis, J. P., Duval, T. S., & Rombach, D. (2001). Personal responsibility for tornado preparedness: Commitment or choice? Journal of Applied Social Psychology, 31(8), 1659-1688.

[25] Gwynne, S., Kuligowski, E. D., & Kinsey, M. (2015). Human behaviour in fire–model development and application. Paper presented at the 6th International Symposium on Human Behaviour in Fire. Interscience Communications.

[26] Kuligowski, E. (2015). Model building: An examination of the pre-evacuation period of the 2001 World Trade Center disaster. Fire and Materials, 39(4), 285-300.

[27] Proulx, G. (2000). Why building occupants ignore fire alarms. Construction Technology Update 42. Ottawa: IRC-NRCC.

[28] Kuligowski, E. (2009). The process of human behavior in fires – NIST Technical Note 1632. Gaithersburg, MD: National Institute of Standards and Technology.

[29] Proulx, G. (2003). Playing with fire: Understanding human behavior in burning buildings. ASHRAE Journal, 45(7), 33-35.

